# Lower charge, higher order: Revising electrostatic control of nematic phases in 2D polyelectrolytes

**DOI:** 10.1073/pnas.2527538123

**Published:** 2026-02-19

**Authors:** Mohsen Moazzami Gudarzi, Mohamad Ali Sanjari Shahrezaei, Seyed Hamed Aboutalebi

**Affiliations:** ^a^National Graphene Institute, University of Manchester, Manchester M13 9PL, United Kingdom; ^b^Department of Physics and Astronomy, School of Natural Sciences, The University of Manchester, Manchester M13 9PL, United Kingdom; ^c^Condensed Matter National Laboratory, Institute for Research in Fundamental Sciences, Tehran 19395-5531, Iran; ^d^School of Quantum Physics and Matter, Institute for Research in Fundamental Sciences, Tehran 19395-5531, Iran

**Keywords:** two-dimensional polyelectrolytes, nematic ordering, graphene oxide, electrostatic screening, structural color

## Abstract

Classical theories of nematic ordering in charged platelets predict that increasing surface charge stabilizes alignment. They also predict enhanced alignment when ionic strength is reduced. Here, in agreement with established theories, we show that in graphene oxide (GO) and related two-dimensional polyelectrolytes, nematic order and structural color emerge as surface charge density is reduced, concomitant with a decrease in self-generated ionic strength and an increase in the effective range of electrostatic repulsion. Using a minimal model combining counterion-only Poisson–Boltzmann electrostatics, van der Waals attraction, and Helfrich undulation, we show that ordering is governed by a self-screened electrostatic length scale, d_EDL_, set by particle-released counterions. When d_EDL_ scale becomes comparable to the interlayer spacing, spacing fluctuations are suppressed and long-range orientational order is enhanced. Our results provide a general framework for controlling structure and optics in 2D polyelectrolyte assemblies.

Long-range ordering in anisotropic colloidal dispersions can yield liquid-crystalline phases that can, in principle, enable coherent light scattering and structural color, similar to crystalline solids. Despite this, highly charged graphene oxide (GO) and related nanosheets rarely exhibit vivid structural color, even under low-ionic-strength conditions where electrostatic repulsion should favor long-range nematic order (1, 2). This persistent mismatch between surface charge density, electrostatic screening, and mesoscopic order points to a missing element in current descriptions of 2D polyelectrolyte liquid crystals.

Here, we show that nematic ordering in two-dimensional polyelectrolytes is enhanced when surface charge is reduced such that the electrostatic screening length (d_EDL_) approaches the interlayer spacing between nanosheets. We demonstrate this principle in GO, where hydrolysis of covalently bound organosulfate groups during purification lowers the effective surface charge density resulting in an increase in EDL thickness. This phenomenon arises from a self-screening mechanism in highly purified GO dispersions, where electrostatic screening is dominated by counterions released from the GO sheets themselves. The resulting increase in d_EDL_ length activates long-range alignment, producing vivid structural color in nematic phases.

## Results

**Fig. 1.**
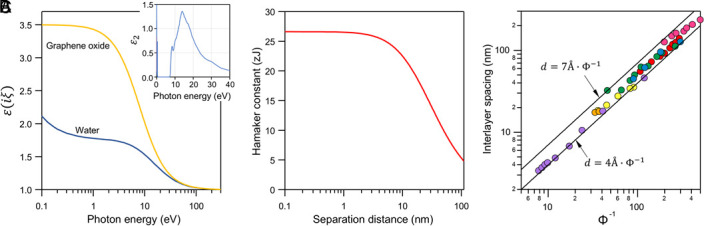
vdW interactions and interlayer spacing in GO dispersions. Panel (*A*) displays the dielectric function of water (3) and graphene oxide (GO) in imaginary frequencies as a function of photon energy. The GO data are computed using the modified harmonic oscillator model, as discussed in the SI. Additionally, Panel (*B*) illustrates the calculated Hamaker constant as a function of the separation distance between GO nanosheets. Panel (*C*) reports average interlayer spacing between GO sheets as a function of reciprocal of volume fraction ($\Phi^{-1}$). The sources of the data are Poulin (orange) (4), Shim (yellow) (6), Xu (green) (7), Shim (blue) (8), Leite Rubim (purple) (9), Li (pink) (10).

We developed a minimal model for the intersheet interactions (*SI Appendix*, section S2). The total disjoining pressure is described by three contributions: i) Poisson–Boltzmann (PB) electrostatics, which depends on surface charge density σ and particle concentration; ii) van der Waals (vdW) attraction, parameterized by a GO–water–GO Hamaker constant calculated from Lifshitz theory (Fig. 1 *A* and *B*) (3, 4); and iii) Helfrich undulation repulsion, capturing entropic fluctuations of flexible sheets (See *SI Appendix* for details). Each GO sheet is treated as a macroion with a large effective valence Z. In this regime, the Debye–Hückel (DH) approximation—i.e., the linearized limit of the PB equation—is invalid because the condition $\left(\frac{Ze\psi_{0}}{k_{B}T}\right)<1$ is not satisfied. The conventional Debye length thus loses its physical meaning and becomes artificially short if GO is treated as a Z:1 electrolyte. Therefore, we estimated the EDL pressure between GO sheets in a salt-free dispersion only accounting for monovalent counterions of GO using the asymptotic form of PB equation at large distance limit as follows (5)

**Fig. 2.**
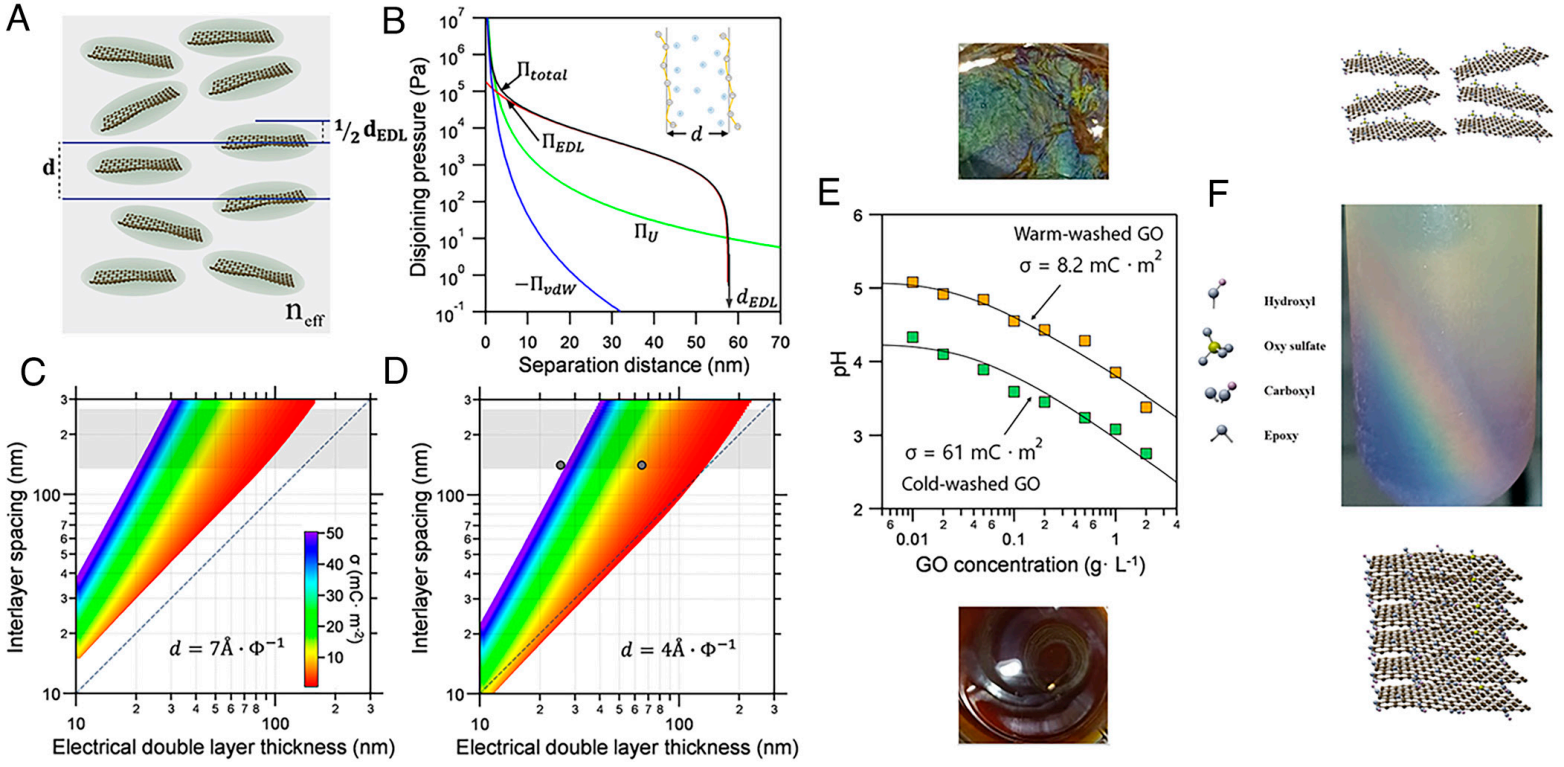
The role of EDL forces on the long-range ordering of GO nanosheets. (*A*) Schematic of long-range ordering of GO nanosheets in a nematic phase, where $d_{\mathrm{EDL}}$ is smaller than interlayer spacing, d. (*B*) Disjoining pressure between GO nanosheets highlights the dominant role of EDL forces over vdW and undulation forces. The critical $d_{\mathrm{EDL}}$ marks the onset of electrostatic repulsion. Panels (*C* and *D*) compare the interlayer spacing d and $d_{\mathrm{EDL}}$ as a function of surface charge density for two values of the packing parameter θ. Gray regions denote spacings that satisfy the first-order Bragg condition for visible color, and dashed lines indicate the estimated threshold for colloidal crystallization. Gray symbols in (*D*) identify samples examined in (*E*). (*E*) The pH data are fitted assuming the protons are mainly counterions of GO with a minor background concentration (11). Notably, GO dispersions washed with cold water (green squares) exhibit lower pH than those washed with warm water (70 °C) at the same solid content. The *Bottom* inset shows a photograph of an aqueous GO dispersion (≈5 g L^−1^) washed with cold water, while the *Top* inset depicts GO nanosheets washed with warm water and redispersed in ethanol (≈5 g L^−1^). (*F*) After high-speed centrifugation, phase separation produces a vertical color gradient: Dilute top layers appear brown–yellow, while denser layers shift from green to blue.

[1] $$\Pi_{\mathrm{EDL}}=\frac{\pi }{2}\frac{k_{B}T}{l_{B}(d+2l_{\mathrm{GC}}{)}^{2}}-k_{B}T(1+Z)c_{\mathrm{GO}},$$

where $l_{B}=\frac{e^{2}}{4\pi \varepsilon_{0}\varepsilon k_{B}T}$ represents the Bjerrum length and $l_{\mathrm{GC}}=\frac{2\varepsilon_{0}\varepsilon k_{B}T}{e\sigma }$ is Gouy–Chapman length. $k_{B}T$ is thermal energy, e is the elementary charge, $\varepsilon_{0}$ the dielectric permittivity of vacuum, ε the dielectric constant of medium, σ is surface charge density of GO, and $c_{\mathrm{GO}}$ is number density of GO sheets. The model does not account for any charge regulation. The factor $(1+Z{)c}_{\mathrm{GO}}$ represents the counterions concentration and is derived from the mass concentration, specific surface area, and σ (*SI Appendix*). We define $d_{EDL}$ as the separation where the total disjoining pressure vanishes, $\Pi_{tot}\left(d_{EDL}\right)=0$ (Fig. 2 *A* and *B* and *SI Appendix*, section S2), and compute $d_{EDL}$ as a function of σ and GO content. We then compare this length scale with interlayer spacing (Fig. 2 *C* and *D*).

An ideal swelling-law predicts $d=\frac{t}{\phi }$, where t is GO thickness (Fig. 1*C*) (12), assuming perfectly parallel stacking and uniform packing. This assumption breaks down for polydisperse GO, which typically forms nematic phases (13). We introduce a packing order factor, θ, as a geometric measure that relates the experimentally observed interlayer spacing d to the ideal perfectly stacked platelet reference at the same volume fraction, such that $d=\frac{t\;\theta }{\phi }$. This formulation accounts for structural heterogeneity in the assemblies. Consistently, experiments report d·ϕ values below the dry GO spacing (~7 Å, Fig. 1*C*).

For structural color, the layered arrangement must satisfy the Bragg condition, $d\approx \frac{\lambda_{\mathrm{vis}}}{2n_{\mathrm{eff}}}$, where $\lambda_{\mathrm{vis}}$ is a visible wavelength and $n_{\mathrm{eff}}$ the medium’s refractive index (gray regions, Fig. 2 *C* and *D*). (2) In nematic GO liquid crystals, the layer spacing measured by SAXS reflects an average across a wide distribution of sheet separations. This uneven spacing smears out the Bragg resonance, yielding a dull or colorless appearance (14). Vivid structural color can only arise when these fluctuations are suppressed—when long-ranged EDL forces span distances comparable to d, locking the layers into a more uniform, optically coherent arrangement.

Our analysis shows that the d_EDL_ becomes comparable to d only when σ is small (<5 mC m^−2^) within the reported range of θ (Fig. 2 *C* and *D*). Interestingly, this contradicts the common perception that stronger EDL repulsion is necessary for structural color formation in GO (10). Our calculations demonstrate that larger σ (and consequently stronger EDL forces) leads to a higher concentration of counterions in the systems, resulting in enhanced screening of the EDL. Given that the Gouy–Chapman length ($l_{\mathrm{GC}}$) is much smaller than $d\approx \frac{\lambda_{\mathrm{vis}}}{2n_{\mathrm{eff}}}$, according to Eq. **1**, it is the osmotic pressure term that controls d_EDL_. Therefore, the effective charge that GO sheets carry, Z, is the dominant factor. In this limit, $l_{\mathrm{GC}}\ll d$, d_EDL_ scales with inverse of square root of counterions concentration and as a result with $\frac{1}{\sqrt{\sigma }}$. We note that at very low σ and in the absence of impurities, crystallization or arrested phases should emerge when $d_{\mathrm{EDL}}\geq d$ and electrostatic repulsion dominates thermal energy; if the repulsion weakens, vdW attractions prevail, leading to aggregation and gelation of the GO dispersion.

Notably, GO dispersions with smaller θ are more likely to host structural color at a given solid content and σ. Although θ could, in principle, be extracted from scattering data, its physical origin and controlling parameters remain elusive (*SI Appendix*). Indeed, small-angle scattering measurements by Shim et al. (6) suggest smaller $\theta =\frac{d\phi }{t}$ for larger GO sheets, consistent with observations of structural color in large-sized GO (10). Thus, *θ* provides a physically meaningful link between particle uniformity, electrostatic interactions, and the emergence of structural color in GO dispersions (*SI Appendix*). Experimentally, we find that reducing σ via controlled hydrolysis of organosulfates, as demonstrated previously (11), is sufficient to trigger nematic alignment and photonic response. Cold-washed dispersions, which retain high sulfur content and consequently high σ, remain optically featureless. By contrast, warm-washed dispersions, where organosulfate groups are mostly removed and σ is reduced, display bright, angle-independent colors (Fig. 2*E*). In concentrated GO dispersions, oxygenated groups are protonated due to the low pH of the dispersion (11). pH measurements confirm that colorful samples possess nearly an order of magnitude fewer acidic groups and therefore lower effective surface charge. Additionally, when warm-washed GO dispersions are subjected to high-speed centrifugation, the resulting phase-separated column displays a vertical rainbow, providing direct visual evidence that photonic response is governed by the interplay between interlayer spacing and electrostatic screening (Fig. 2*F*). The dilute top layers appear brownish-yellow, while increasingly packed lower layers shift through green into blue as interlayer spacing decreases. This gradient reflects the Bragg condition ($d\approx \frac{\lambda_{\mathrm{vis}}}{2n_{\mathrm{eff}}}$); as ϕ increases with depth, d contracts and the reflected wavelength shifts across the visible spectrum.

## Discussion

Our results reveal that in highly purified GO dispersions, and more generally in anisotropic colloids, such as two-dimensional polyelectrolytes, with minimal extrinsic ions, electrostatic screening is governed primarily by counterions released from the particles themselves. Each GO sheet therefore behaves as a macroion with a large effective valence, placing the system in a self-screened regime where the electrostatic screening length increases as surface charge density decreases. Coupled with GO surface chemistry, extensive hydrolysis of sulfate groups—the dominant charge carriers—thus increases $d_{EDL}$, allowing orientational order to propagate and giving rise to structural color.

The universality of the $d_{\mathrm{EDL}}$ ≥ d criterion explains the strong sensitivity of liquid-crystalline order to preparation, ionic environment, and solvent choice. This design rule also suggests a practical lever for LCGO wet spinning, as partial decharging can increase $d_{EDL}$ and strengthen long-range registry at spinning concentrations, potentially improving alignment coherence prior to coagulation. These results revise the classical view of charge-controlled liquid crystallinity and enable tuning of structure and optics via surface chemistry.

## Materials and Methods

GO was synthesized using a modified Hummers’ method, following our previous reports (13). Washing/purification was performed at either room temperature or 70 °C to modulate surface charge. Intersheet interactions were modeled as the sum of counterion-only Poisson–Boltzmann electrostatic pressure, van der Waals interactions (Hamaker constant from Lifshitz theory with retardation), and Helfrich undulation pressure. Full experimental and modeling details are provided in the *SI Appendix*.

## Supplementary Material

Appendix 01 (PDF)

## Data Availability

There are no data underlying this work.

## References

[1] P. Davidson, C. Penisson, D. Constantin, J.-C.P. Gabriel, Isotropic, nematic, and lamellar phases in colloidal suspensions of nanosheets. PNAS **115**, 6662–6667 (2018). 29891691 10.1073/pnas.1802692115 PMC6042086

[2] P. H. Michels-Brito , Bright, noniridescent structural coloration from clay mineral nanosheet suspensions. Sci. Adv. **8**, eabl8147 (2022). 35080971 10.1126/sciadv.abl8147 PMC8791460

[3] M. Moazzami Gudarzi, S. H. Aboutalebi, Self-consistent dielectric functions of materials: Toward accurate computation of Casimir–van der Waals forces. Sci. Adv. **7**, eabg2272 (2021). 34039608 10.1126/sciadv.abg2272 PMC8153719

[4] P. Poulin , Superflexibility of graphene oxide. Proc. Natl. Acad. Sci. **113**, 11088–11093 (2016). 27647890 10.1073/pnas.1605121113 PMC5056031

[5] M. Moazzami-Gudarzi , Interplay between depletion and double-layer forces acting between charged particles in solutions of like-charged polyelectrolytes. Phys. Rev. Lett. **117**, 088001 (2016). 27588884 10.1103/PhysRevLett.117.088001

[6] Y. H. Shim, E. H. Cho, S. Y. Kim, Unifying dispersion properties of graphene oxide suspensions via interlayer spacing control: Insights for universal 2D colloid behavior. Carbon **215**, 118473 (2023).

[7] Z. Xu, C. Gao, Graphene chiral liquid crystals and macroscopic assembled fibres. Nat. Commun. **2**, 571 (2011). 22146390 10.1038/ncomms1583 PMC3247827

[8] Y. H. Shim, K. E. Lee, T. J. Shin, S. O. Kim, S. Y. Kim, Wide concentration liquid crystallinity of graphene oxide aqueous suspensions with interacting polymers. Mater. Horiz. **4**, 1157–1164 (2017).

[9] R. Leite Rubim , Highly confined stacks of graphene oxide sheets in water. Eur. Phys. J. E **41**, 1–10 (2018). 29546498 10.1140/epje/i2018-11636-5

[10] P. Li , Tunable lyotropic photonic liquid crystal based on graphene oxide. ACS Photonics **1**, 79–86 (2014).

[11] M. Moazzami Gudarzi, M. A. Sanjari Shahrezaei, M. Hosseini, S. H. Aboutalebi, Redefining graphene oxide: The role of organosulfate groups in charging dynamics and colloidal stability. Small Struct. **6**, 2500035 (2025).

[12] Y. H. Shim, H. Ahn, S. Lee, S. O. Kim, S. Y. Kim, Universal alignment of graphene oxide in suspensions and fibers. ACS Nano **15**, 13453–13462 (2021). 34324294 10.1021/acsnano.1c03954

[13] S. H. Aboutalebi, M. M. Gudarzi, Q. B. Zheng, J.-K. Kim, Spontaneous formation of liquid crystals in ultralarge graphene oxide dispersions. Adv. Funct. Mater. **21**, 2978–2988 (2011).

[14] G. Shang, M. Eich, A. Petrov, Photonic glass based structural color. APL Photon. **5**, 060901 (2020).

