# Supplementary material for: Lower charge, higher order: Revising electrostatic control of nematic phases in 2D polyelectrolytes
Source: Proc Natl Acad Sci U S A. 2026 Feb 19;123(8):e2527538123. doi: 10.1073/pnas.2527538123 (PMC12933090; doi:10.1073/pnas.2527538123)
Supplement: Supplementary file 1 — Appendix 01 (PDF) [file pnas.2527538123.sapp.pdf]

Supporting Information for

Lower charge, higher order: revising electrostatic control of nematic phases in 2D polyelectrolytes

Mohsen Moazzami Gudarzi [a, b], Mohamad Ali Sanjari Shahrezaei [c], and Seyed Hamed Aboutalebi [c, d]

[a] National Graphene Institute, University of Manchester, Manchester, M13 9PL UK

[b] Department of Physics and Astronomy, School of Natural Sciences, The University of Manchester, Manchester, M13 9PL UK

[c] Condensed Matter National Laboratory, Institute for Research in Fundamental Sciences (IPM), Tehran, 19395-5531, Iran

[d] School of Quantum Physics and Matter, Institute for Research in Fundamental Sciences (IPM), Tehran, 19395-5531, Iran

*Mohsen Moazzami Gudarzi, Seyed Hamed Aboutalebi

Email: mohsen.moazzamigudarzi@manchester.ac.uk, hamedaboutalebi@ipm.ir

This PDF file includes:

Supporting text
SI References

Supporting Information Text

S1. Materials preparation:

Graphene oxide (GO) was synthesized using a modified Hummers' method following our previous reports.(1-5) Briefly, 0.5 g of graphite flakes (3772, Asbury Graphite Mills, US) was placed in a 250 mL glass vessel immersed in an ice bath. To this, 50 mL of concentrated sulfuric acid (98%, Chemlab) was added, followed by the gradual addition of 3.5 g of potassium permanganate (Scharlau) under vigorous stirring. The mixture was left to react overnight. Neutralization was initiated by the slow, dropwise addition of 50 mL of deionized (DI) water (Millipore, 25 °C) under high-shear mixing, while maintaining the vessel in an ice bath to avoid overheating. Residual permanganate was fully quenched by adding 7 mL of hydrogen peroxide (30%, Merck), pre-diluted in 13 mL of DI water at 25 °C. The resulting suspension was then subjected to three washing cycles with 1 M HCl (prepared by diluting 37% fuming HCl, Merck, in DI water at 25 °C), using centrifugation and re-dispersion. The final suspension was then subjected to repeated washing steps by DI water (at 25 °C), and 70 °C).

S2. Modeling interactions between two GO sheets:

The total pressure (Π_t) between two adjacent GO sheets is assumed to be the sum of three main forces, namely electrical double layer (EDL), van der Waals (vdW) and undulation (U) forces:

$$\Pi_t = \Pi_{EDL} + \Pi_{vdW} + \Pi_U. \qquad \text{(S1)}$$

We ignored hydration forces which are only significant at very short distances.

S2. 1. *Self-Screening and Electrostatic Interactions in Purified GO Dispersions*

In dispersions containing only GO, the protons dissociated from GO are the only species screening the EDL. In this "*self-screened*" regime, each GO sheet behaves as a macro-ion with a large effective valence (Z), and the Debye–Hückel approximation becomes invalid because the condition ($\frac{Ze\psi_0}{k_B T}$) <1 is not satisfied (DH model is the linearized approximation of the Poisson–Boltzmann (PB) model and is only valid at low surface potential)(6). The conventional Debye length thus loses its usual meaning or becomes unrealistically short if treated as a Z:1 electrolyte (7).

Within the DH framework, the exponential decay of electrostatic repulsion implies a finite, long-ranged interaction for extended objects such as nanosheets, whose large interacting area can sustain repulsion over distances (significantly larger than Debye length) where the interaction energy remains comparable to thermal energy. To reconcile this with experimental observations of finite interlayer spacings, several studies supplement DH electrostatics with an attractive van der Waals (vdW) interaction, which follows a power-law decay and can therefore introduce a secondary minimum in the effective interaction potential at distances much larger than the nominal Debye length (8-10). We note that these analyses often neglect retardation effects in vdW interactions, which become important at the length scales relevant to structural colour; (11)including retardation would be expected to shift the location of any such minimum to even larger separations.

The interpretation commonly adopted in these works is that the average interparticle spacing measured by X-ray scattering and/or optical reflection corresponds to this secondary minimum, implying that nanosheets are effectively localized by an energy trap. While such an approach may be reasonable in certain parameter regimes, we believe it is incomplete for highly purified GO dispersions. In particular, a picture based on particles trapped in a well-defined secondary minimum would more naturally be associated with positional ordering or phase separation, whereas experiments on purified GO typically reveal nematic order with broad scattering features rather than sharp Bragg peaks (12-14). Factors such as polydispersity, orientational entropy, and thermal fluctuations may further smear out any putative energy minimum, making this interpretation less compelling in the present context.

Alternative treatments sometimes attempt to modify excluded-volume interactions by adding an *ad hoc* multiple of the Debye length to the effective nanosheet thickness (15), a notion that goes back to Onsager himself (16). However, these approaches face a fundamental difficulty: in concentrated, highly purified nanosheet dispersions, the ionic strength is ill-defined. Estimating a Debye length based solely on residual background ions does not capture the dominant screening mechanism when counterions released by the particles themselves control the electrostatics.

To properly describe this regime, we adopt the counterion-only solution of the PB equation, following the formulation introduced in ref.(17) and validated experimentally for highly

charged colloids. To this end, the PB equation, at large separation distance (d), can be analytically approximated(17):

$$\Pi_{EDL} = \frac{\pi}{2}\frac{k_B T}{l_B(d+2l_{GC})^2} - k_B T(1+Z)c_{GO}. \quad (S2)$$

Where $k_B T$ is thermal energy, $l_B = \frac{e^2}{4\pi\varepsilon\varepsilon_0 k_B T}$ is the Bjerrum length and $l_{GC} = \frac{2\varepsilon\varepsilon_0 k_B T}{e\sigma}$ is the Gouy-Chapman length. ε_0, ε, σ are vacuum permittivity, medium dielectric constant and surface charge density, respectively. Z is the effective charge of GO sheets and c_{GO} is particle concentration of GO. The term $(1+Z)c_{GO}$ denotes the counterion concentration, which follows from charge neutrality and is set by the GO surface charge density σ, specific surface area A, and mass concentration m, according to

$$(1+Z)c_{GO} = \sigma \cdot A \cdot m. \quad (S3)$$

The GO surface area is taken as 1700 $m^2 \cdot g^{-1}$, consistent with our previous work (18).

S2.2. Non-Electrostatic Interactions: vdW and Undulation Pressures

We approximated vdW forces between two GO sheets using the expression for two plates of thickness of t interacting at separation distance of d (19):

$$\Pi_{vdW} = -\frac{H}{6\pi}\left(\frac{1}{d^3} + \frac{1}{(d+2t)^3} - \frac{2}{(d+t)^3}\right), \quad (S4)$$

Where H represents the Hamaker constant, estimated from the Lifshitz theory accounting for retardation effect (19):

$$H(d) = \frac{3k_B T}{2}\sum_{n=1}^{\infty} R_n(d)\sum_{s=1}^{\infty}\frac{\Delta^{2s}}{s^3}, \quad (S5)$$

$$\Delta = \frac{\varepsilon_{GO}(i\xi_n) - \varepsilon_{water}(i\xi_n)}{\varepsilon_{GO}(i\xi_n) + \varepsilon_{water}(i\xi_n)}, \quad (S6)$$

Where ε_i represents the dielectric function at imaginary frequencies (Figure 1A) and

$$\xi_n = \frac{2\pi n k_B T}{\hbar}, \quad (S7)$$

$$R_n(d) = \left(1 + \frac{2d\xi_n\sqrt{\varepsilon_{water}(i\xi_n)}}{c}\right)e^{-\frac{2d\xi_n\sqrt{\varepsilon_{water}(i\xi_n)}}{c}}, \quad (S8)$$

Where c is speed of light and $\hbar$ is the reduced Planck constant. Note that we ignored the zero-term frequency in our calculations as this term is small and is mostly screened in the presence of salts(19). We used the experimental optical constants of water compiled in our previous work to compute ε_{water} (Figure S5A).(11) For GO, we used the modified harmonic oscillator model introduced in our previous work.(11) We ignored optical

anisotropy and bands in infrared regions. The electronic dielectric constant and optical band gap of GO is approximated from Schwenzer *et al.* to be 3.5, 3.7 eV, respectively.(20) The density and the chemical formula of GO is estimated as C_5O_2 and 1.8 g·cm^{-3}, respectively.(21) We found non-retarded Hamaker constant of ≅ 27×10^{-21} J (Figure 1B). The undulation pressure is approximated using the expression derived by Helfrich (22):

$$\Pi_U = \frac{3\pi^2(k_B T)^2}{64\kappa_b d^3}, \qquad \text{(S9)}$$

Where κ_b represents the bending modulus of GO approximately equal to $k_B T$ (13).

S2.3. Definition and Scaling of the EDL Thickness

We approximated the thickness of the double layer (d_{EDL}) around GO for a given concentration using the following equation:

$$\Pi_t(d_{EDL}) = 0. \qquad \text{(S10)}$$

We then compared the d_{EDL} obtained from above model with the interlayer layer spacing of GO sheets at different volume fraction (Figures 2D,E). Within this framework, the σ sets both the strength and range of electrostatic repulsion. When the l_{GC} is much smaller than the interparticle spacing (d), d_{EDL} scales as the inverse square root of the counterion concentration—and thus of the surface charge density—analogous to the Debye length in low-valence electrolytes. (See eq. S2) Extending this analogy by treating the counterion concentration as the ionic strength, eq. S2 predicts an EDL thickness that is 2π times larger than the corresponding Debye length. Note that the interlayer spacing depends on the packing order factor, θ, which can be estimated from the experimental data, $\theta = \frac{\phi d}{t}$ (Figures 1C).

The above argument suggests that tuning the surface charge density σ offers a direct and effective route to control the EDL thickness d_{EDL}. In purified GO dispersions, we have shown that the dominant source of surface charge is organosulfate groups, whose density can be systematically adjusted through controlled hydrolysis (18). Alternatively, σ can be modulated by changing the dielectric constant ε of the medium, as the protonation equilibrium of surface groups is highly sensitive to ε; lowering ε typically leads to a substantial reduction in σ (23). Consequently, solvents with lower ε may support structural

colour more efficiently than water, provided colloidal stability is maintained. Consistent with this, we observe pronounced structural colour when GO is dispersed in ethanol.

S2.4. Packing Disorder and Structural Colour

Furthermore, our calculations indicate that a small packing order factor θ is necessary to observe structural colour. From the reported average interlayer spacing between GO sheets d as a function of volume fraction ϕ this factor can be directly derived; $\theta = \frac{\phi d}{t}$. We define θ as a geometrical measure that compares the experimentally observed interlayer spacing d to the spacing expected for the perfectly stacked platelet reference at the same volume fraction. In the idealized limit of perfectly shaped disks arranged in a fully registered layered geometry, the interlayer spacing is given by $d = t/\phi$, where $t$is the disk thickness and $\phi$the volume fraction. Deviations from this reference arise when disk shapes are irregular and/or when positional registry between neighboring platelets is absent. In the nematic state studied here, orientational order emerges from excluded-volume interactions, while the platelet centers of mass remain spatially disordered and no smectic or lamellar liquid-crystal phase is formed. Under these conditions, the measured spacing $d$can deviate from t/ϕ, reflecting departures from the ideal layered reference geometry. The packing factor θthus quantifies this geometric deviation and serves as an empirical indicator of nematic organization, without being equivalent to a conventional orientational order parameter.

The experimental data (Figure 1C) suggests θ is around 0.8 to 0.6 assuming $t \approx 7$Å for monolayer GO and perfect exfoliation of GO into single layer. The parameters affecting the packing GO (and other layered colloids) is not well established. The problem of the packing regimes of uniform non-spherical particles even with hard-sphere interaction and small aspect ratio becomes very complicated and is still being investigated (24-26). The case of flexible sheets with high aspect ratio interacting with soft EDL forces is therefore not trivial. Given the random nature of oxidative cutting of graphite (27), the size and shape of GO is random. However, the polydispersity in size can be controlled by cascade centrifugation for instance. Interestingly, studies reporting structural colour in GO have observed it in samples with narrower size distributions but larger lateral sizes. (28-30) Indeed, small angle X-ray scattering data by Shim *et al.* suggest larger GO sheets show

smaller packing order factor ($\frac{\phi d}{t}$). (31) Recent work by Kikuchi *et al.(32)* on structural colour in titanate nanosheets reports enhanced colour purity in dispersions with much smaller packing order factor, supporting our hypothesize on the crucial role of packing order on long ranged ordering of 2D colloids.

Usually smaller polydispersity leads to decreased packing of spherical particles, (33, 34) but extension of this conjecture to 2D colloids requires further investigations. Regardless, our findings and analysis reinforce the importance of both a small packing order factor and a low surface charge density for the observation of structural color in 2D colloids.

Acknowledgements

Instrumental characterization and materials preparation facilities were provided by the Condensed Matter National Laboratory at the Institute for Research in Fundamental Sciences (IPM) in Tehran, Iran. Additionally, financial support for equipment purchase was granted by the INSF under project number 4022382.

SI References

1. M. A. Sanjari Shahrezaei *et al.*, Anomalously low electrostatic bending stiffness of graphene oxide 2D membranes regulates their environmental fate in aquatic ecosystems. *Journal of Materials Chemistry A* **10**, 1414-1424 (2022).
2. S. H. Aboutalebi *et al.*, High-Performance Multifunctional Graphene Yarns: Toward Wearable All-Carbon Energy Storage Textiles. *ACS Nano* **8**, 2456-2466 (2014).
3. S. H. Aboutalebi *et al.*, Comparison of GO, GO/MWCNTs composite and MWCNTs as potential electrode materials for supercapacitors. *Energy & Environmental Science* **4**, 1855-1865 (2011).
4. S. H. Aboutalebi, M. M. Gudarzi, Q. B. Zheng, J.-K. Kim, Spontaneous Formation of Liquid Crystals in Ultralarge Graphene Oxide Dispersions. *Advanced Functional Materials* **21**, 2978-2988 (2011).
5. R. Jalili *et al.*, Silicon as a ubiquitous contaminant in graphene derivatives with significant impact on device performance. *Nature Communications* **9**, 5070 (2018).
6. Anonymous, "Copyright" in Intermolecular and Surface Forces (Third Edition)*,* J. N. Israelachvili, Ed. (Academic Press, San Diego, 2011), https://doi.org/10.1016/B978-0-12-375182-9.10026-0, pp. iv.

7. M. Moazzami-Gudarzi, P. Maroni, M. Borkovec, G. Trefalt, Depletion and double layer forces acting between charged particles in solutions of like-charged polyelectrolytes and monovalent salts. *Soft Matter* **13**, 3284-3295 (2017).
8. K. Sano *et al.*, Photonic water dynamically responsive to external stimuli. *Nature Communications* **7**, 12559 (2016).
9. T. Lan *et al.*, Collective Behavior Induced Highly Sensitive Magneto-Optic Effect in 2D Inorganic Liquid Crystals. *Journal of the American Chemical Society* **143**, 12886-12893 (2021).
10. K. Sano *et al.*, Propagating wave in a fluid by coherent motion of 2D colloids. *Nature Communications* **12**, 6771 (2021).
11. M. Moazzami Gudarzi, S. H. Aboutalebi, Self-consistent dielectric functions of materials: Toward accurate computation of Casimir–van der Waals forces. *Science Advances* **7**, eabg2272 (2021).
12. P. Davidson, C. Penisson, D. Constantin, J.-C. P. Gabriel, Isotropic, nematic, and lamellar phases in colloidal suspensions of nanosheets. *Proceedings of the National Academy of Sciences* **115**, 6662-6667 (2018).
13. P. Poulin *et al.*, Superflexibility of graphene oxide. *Proceedings of the National Academy of Sciences* **113**, 11088-11093 (2016).
14. Z. Xu, C. Gao, Graphene chiral liquid crystals and macroscopic assembled fibres. *Nature Communications* **2**, 571 (2011).
15. D. van der Beek, H. N. W. Lekkerkerker, Liquid Crystal Phases of Charged Colloidal Platelets. *Langmuir* **20**, 8582-8586 (2004).
16. L. Onsager, THE EFFECTS OF SHAPE ON THE INTERACTION OF COLLOIDAL PARTICLES. *Annals of the New York Academy of Sciences* **51**, 627-659 (1949).
17. M. Moazzami-Gudarzi *et al.*, Interplay between Depletion and Double-Layer Forces Acting between Charged Particles in Solutions of Like-Charged Polyelectrolytes. *Physical Review Letters* **117**, 088001 (2016).
18. M. Moazzami Gudarzi, M. A. Sanjari Shahrezaei, M. Hosseini, S. H. Aboutalebi, Redefining Graphene Oxide: The Role of Organosulfate Groups in Charging Dynamics and Colloidal Stability. *Small Structures* **6**, 2500035 (2025).
19. V. A. Parsegian, *Van der Waals forces: a handbook for biologists, chemists, engineers, and physicists* (Cambridge University Press, 2005).
20. B. Schwenzer, T. C. Kaspar, Y. Shin, D. W. Gotthold, Spectroscopic Study of Graphene Oxide Membranes Exposed to Ultraviolet Light. *The Journal of Physical Chemistry C* **120**, 12559-12567 (2016).
21. M. M. Gudarzi, Colloidal Stability of Graphene Oxide: Aggregation in Two Dimensions. *Langmuir* **32**, 5058-5068 (2016).
22. W. Helfrich, Steric interaction of fluid membranes in multilayer systems. *Zeitschrift für Naturforschung A* **33**, 305-315 (1978).
23. G. Kokot, M. I. Bespalova, M. Krishnan, Measured electrical charge of SiO2 in polar and nonpolar media. *The Journal of Chemical Physics* **145** (2016).
24. A. Donev *et al.*, Improving the Density of Jammed Disordered Packings Using Ellipsoids. *Science* **303**, 990-993 (2004).
25. L.-N. Zou, X. Cheng, M. L. Rivers, H. M. Jaeger, S. R. Nagel, The Packing of Granular Polymer Chains. *Science* **326**, 408-410 (2009).

26. C. Ness, R. Seto, R. Mari, The Physics of Dense Suspensions. *Annual Review of Condensed Matter Physics* **13**, 97-117 (2022).
27. P. M. Ajayan, B. I. Yakobson, Oxygen breaks into carbon world. *Nature* **441**, 818-819 (2006).
28. P. Li *et al.*, Tunable lyotropic photonic liquid crystal based on graphene oxide. *ACS photonics* **1**, 79-86 (2014).
29. D. Ogawa, T. Nishimura, Y. Nishina, K. Sano, A magnetically responsive photonic crystal of graphene oxide nanosheets. *Nanoscale* 10.1039/D3NR06114K (2024).
30. T.-Z. Shen, S.-H. Hong, B. Lee, J.-K. Song, Bottom-up and top-down manipulations for multi-order photonic crystallinity in a graphene-oxide colloid. *NPG Asia Materials* **8**, e296-e296 (2016).
31. Y. H. Shim, E. H. Cho, S. Y. Kim, Unifying dispersion properties of graphene oxide suspensions via interlayer spacing control: Insights for universal 2D colloid behavior. *Carbon* **215**, 118473 (2023).
32. T. Kikuchi *et al.*, Lateral Size Modulated Structural Color of Aqueous Dispersions of Titanate Nanosheets. *Chemical Communications* 10.1039/D5CC06149K (2026).
33. R. S. Farr, R. D. Groot, Close packing density of polydisperse hard spheres. *The Journal of chemical physics* **131** (2009).
34. F. Zamponi, Packings close and loose. *Nature* **453**, 606-607 (2008).
